# sEMG-Based Drawing Trace Reconstruction: A Novel Hybrid Algorithm Fusing Gene Expression Programming into Kalman Filter

**DOI:** 10.3390/s18103296

**Published:** 2018-09-30

**Authors:** Zhongliang Yang, Yangliang Wen, Yumiao Chen

**Affiliations:** 1 College of Mechanical Engineering, Donghua University, Shanghai 201620, China; yzl@dhu.edu.cn (Z.Y.); wenyangliang@outlook.com (Y.W.); 2 School of Art, Design and Media, East China University of Science and Technology, Shanghai 200237, China

**Keywords:** drawing trace, electromyography, gene expression programming, Kalman Filter, muscle-computer interface

## Abstract

How to reconstruct drawing and handwriting traces from surface electromyography (sEMG) signals accurately has attracted a number of researchers recently. An effective algorithm is crucial to reliable reconstruction. Previously, nonlinear regression methods have been utilized successfully to some extent. In the quest to improve the accuracy of transient myoelectric signal decoding, a novel hybrid algorithm KF-GEP fusing Gene Expression Programming (GEP) into Kalman Filter (KF) framework is proposed for sEMG-based drawing trace reconstruction. In this work, the KF-GEP was applied to reconstruct fourteen drawn shapes and ten numeric characters from sEMG signals across five participants. Then the reconstruction performance of KF-GEP, KF and GEP were compared. The experimental results show that the KF-GEP algorithm performs best because it combines the advantages of KF and GEP. The findings add to the literature on the muscle-computer interface and can be introduced to many practical fields.

## 1. Introduction

As computers grow more powerful and widely available, a number of user-interface researchers are seeking ways to achieve natural human-computer interaction (HCI) more accessible. Recently, an interface that converts human bio-electric activity to external devices, especially the muscle-computer interface (MuCI), has become a new research focus in HCI.

The new interaction style MuCI is mediated by physiological data [1], that is, surface electromyography (sEMG) signals [2]. Once the impulses of activation of the innervating motor neurons [3] are triggered, the sEMG will register the electrical activity of the muscle fibers during a motor task in a non-invasive way. Recently, a variety of sEMG-based interfaces has been developed for hand gesture recognition [4], prosthesis control [5,6], sign languages recognition [7] and angle recognition of upper limbs [8]. Therefore, after 60 years of development [9], discrete symbol recognition from sEMG signals has been known as a relatively easy task. Additionally, a further challenging task appears with demands for a novel and natural interactive paradigm that enables people to be more creative, expressive and satisfied in their daily lives, for instance, to reconstruct continuous drawing traces from sEMG signals.

Several methods have been proposed for the automatic recognition and reconstruction of drawing traces. Among these methods, the most typically familiar and traditional way is to record pictured characters with the pen, touch medium tablet, touchscreen or mouse and synchronously transmit drawing trace messages to computer. Many of the most robust recognition systems have been widely used for gesture or isolated symbol recognition [10], such as feature-based statistical classifiers [11], ad-hoc heuristic recognizers [12], $1 recognizer [13] and the incremental recognition algorithm [14]. However, these traditional interfaces cannot transmit drawing traces produced in the air. The recent achievement in computer vision offered a way to recognize drawing traces in the mid-air [15,16]. However, when the input images or videos are not high-quality which are vulnerably related with such factors as camera angle, background and lighting [17,18], it occurs that attaining high recognition accuracy using the computer-vision based methods is not satisfied. These disadvantages of the computer-vision based methods can be avoided by the sEMG-based methods.

The existing research has demonstrated the feasibility of decoding hand-written traces solely from sEMG signals [19]. The recognition performance attained with the sEMG-based methods is comparable to that achieved by computer-vision based methods of written character recognition [16,20,21,22,23]. Besides, several researchers have considered solving the problem of handwriting traces reconstruction from sEMG activity, which involved the application of regression techniques for hand-written traces reconstruction from sEMG, such as Wiener filter and the Kalman filter (KF). However, the average accuracy of the Wiener filter [19] is 47% for *x*-coordinate and 63% for *y*-coordinate. After developed by the KF [3], the average accuracy reaches 63% for *x*-coordinate and 73% for *y*-coordinate.

In conclusion, since the study on drawing trace reconstruction from sEMG signals is still in its rudimentary stage, a set of advanced algorithms for dynamic trace representation, sEMG feature extraction, kinematic velocity transition, sEMG classification and continuous decoding are urgently needed to achieve high reconstruction performance.

In our previous study [24], we proposed a three-step hybrid model based on Gene Expression Programming (GEP) for the reconstruction of hand-drawn shapes from multichannel sEMG signals. Although the results showing that this GEP model slightly outperforms the KF, we can still consider the temporal and spatial context properties of drawing through KF to improve the reconstruction performance of GEP model.

This study proposes a hybrid method fusing the GEP into the KF framework for the sEMG-based drawing trace reconstruction. The main idea behind our method is to replace the measurement transformation matrix of the observation model and the covariance of the observation noise in the KF with the GEP models. Five participants volunteered to attend the experiments. They were required to draw on a digital tablet with a pen. Meanwhile, the sEMG signals were recorded from arm muscles. There were two groups of symbols selected to verify the validity of our method, a group including 14 basic one-stroke shapes and the other containing 10 numeric characters from 0 to 9.

## 2. Materials and Methods

### 2.1. The Experiment

#### 2.1.1. Participants

The experiments were conducted under the approval of the Ethics Review Committee of Donghua University. We recruited five male volunteers at the age of 25–33, 169–178 cm in height, 62–73 kg in weight for this study, while all the participants would be medically examined to make sure that they had no upper limb musculoskeletal and nervous diseases and they were all right-handed. In addition, any intense upper-limb activity was not allowed before the experiment to avoid muscle fatigue.

#### 2.1.2. Drawn and Written Symbols

**Figure 1:**
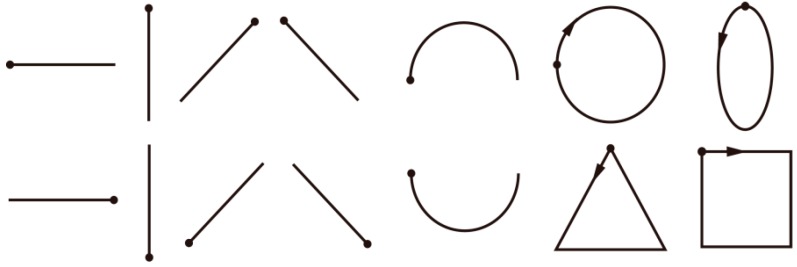
The fourteen basic one-stroke hand-drawn shapes used in our study. Dots represent starting points, arrows represent directions. The shapes are, from left to right, in order, horizontal line, vertical line, forward slash, backslash, arch, circle, ellipse, reversed horizontal line, reversed vertical line, reversed forward slash, reversed backslash, reversed arch, square and triangle.

From the existing study, we know that each multi-stroke drawing and handwriting, either in a simple or complicated way, can be produced with an arbitrary number of single strokes, considered as a primitive of handwriting and drawing [25]. We selected 14 basic widely-used one-stroke shapes which are not only for simplicity but also corresponded with the request of diversity with different shapes and angles during drawing in this exploratory experiment. Figure 1 shows the images of fourteen basic one-stroke hand-drawn shapes used.

**Figure 2:**
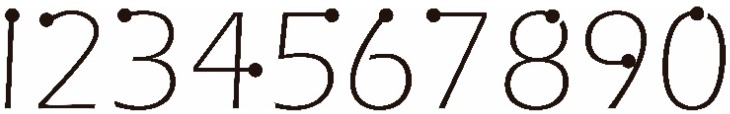
The ten numeric characters used in our study. Dots represent starting points.

Since no literature have been published to provide data on EMG-drawing reconstruction, we cannot find some other drawing reconstruction methods for comparison. In order to verify the validity of our method, we selected another group of written characters for research, containing numeric characters from 0 to 9. Figure 2 shows the images of ten one-stroke numeric characters used.

#### 2.1.3. Test Muscles

Eight channels were set to record sEMG activities in this study. One channel measuring the sEMG activities of thumb over Adductor Pollicis was set as the trigger channel to discriminate the drawing onset. The channel was set to trigger the coordinate recording of the digital tablet. The feature extraction of sEMG signals from the remaining seven channels were triggered simultaneously. The remaining seven channels were set to record sEMG signals over four forearm muscles: the Extensor Carpi Radialis Brevis, Extensor Carpi Ulnaris, Extensor Digitorum, Flexor Carpi Radialis and three upper arm muscles: Biceps Brachii, Deltoid Muscle and Triceps Brachii. For a detailed illustration of the locations of the electrodes, see [24].

#### 2.1.4. Experimental Protocol

There were two sources of data collected simultaneously in the experiments. One was the recording of drawing traces by digital tablet, yielding *X*-coordinates and *Y*-coordinates. The other one was the recording of sEMG signals from electrodes attached to the skin overlying muscles.

**Figure 3:**
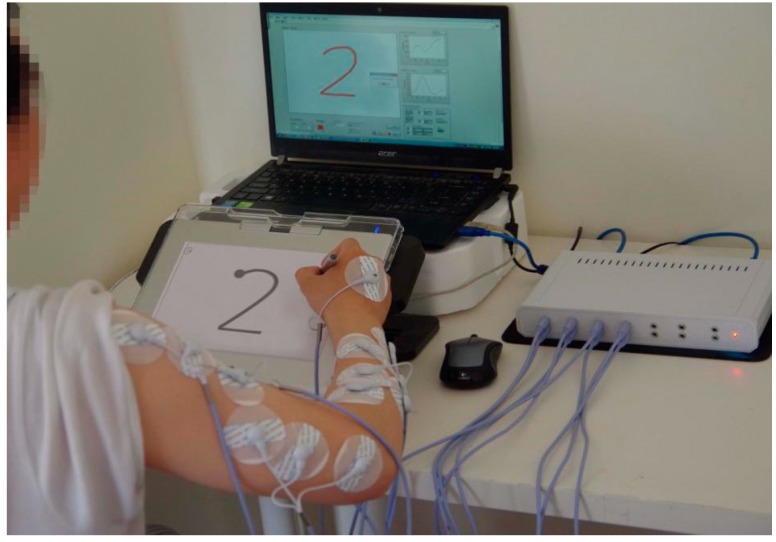
A photograph of one subject in the task stage.

An introduction phase, a preparation phase and a task phase were needed in the experiment. In the introduction phase, the equipment and procedures, tasks and risks were introduced to the subjects. They were instructed to sign a consent form after anthropometric measurements. In the preparation phase, all selected shapes were printed on 120 × 120 mm^2^ paper as templates placed on the work area of the digital tablet with transparent photo frame cover. Twenty-four sheets of template paper were prepared for the task phase. In the task phase, the sEMG electrodes were attached to the right arm and hand of one subject. After all the signals showed stable and normal, the subject was required to sit at a desk in front of the digital tablet with a comfortable posture, as shown in Figure 3. Then he clicked and held the starting button of the digital pen to trigger the drawing trace collection in terms of *X*-coordinates and *Y*-coordinates. At the same time, the sEMG signals were recorded synchronously. When the shape was drawn, he should release the starting button to end the trace collection.

The subject traced and repeated each symbol approximately 40 times. The experimenter replaced the template paper in a random order. In total, 960 trials were performed by each participant for two days. On the first day, the subject was requested to finish the drawing session. The handwriting session was carried out on the following day. After 10 consecutive trials, the subject had a rest for 3 min to avoid muscle fatigue. During rest periods, the experimenter replaced the template randomly. In general, it took each participant approximately 800 min to finish the experiment.

#### 2.1.5. Data Acquisition

**Figure 4:**
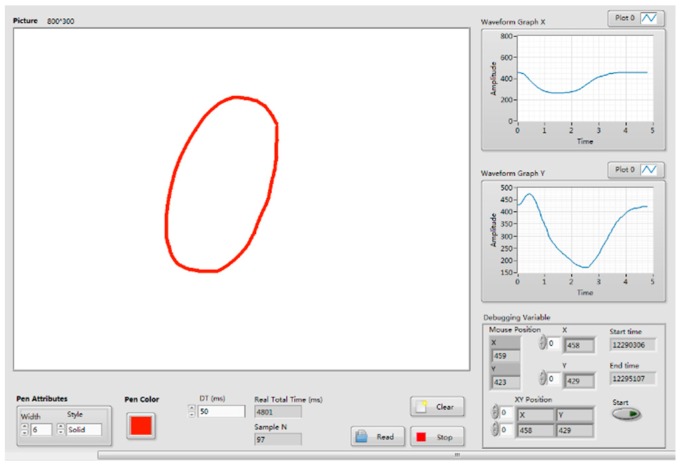
The Drawing Coordinates Acquisition System (DCAS) software developed in Labview.

There were two sources of acquired data in our system: *X* and *Y* coordinates of drawing traces and sEMG signals. The *X* and *Y* coordinates were recorded and visualized by the self-developed software, that is, the Drawing Coordinates Acquisition System (DCAS), as shown in Figure 4. The DCAS can capture the *X* and *Y* coordinates of traces produced by the digital pen in the drawing area. The sampling frequency can be adjusted to be in accordance with that of sEMG signals.

The sEMG signals acquisition scheme, experimental instruments, sensors and electrodes are the same as those used in Reference [24]. The raw sEMG signals were sampled at 1000 Hz and band-pass filtered at 10–500 Hz with a notch filter implemented to remove the 50 Hz line interference. The trigger channel was used to determine the onset of each trial. The remaining channels were used to further extract features. Once the threshold crossing was detected by the trigger channel, its epoch onset would be designated for the remaining seven channels. We set the threshold of sEMG amplitude as 30 µV. After the first amplitude value crossing the threshold, the moment of the maximum peak value in the coming 20 ms can be defined as the epoch onset.

#### 2.1.6. Two Experimental Designs

Two basic experiments designed to calibrate our reconstruction model were conducted in this study. For within-group reconstruction, we built one single set of reconstruction models (F(*x*) and F(*y*)) from all shapes in one dataset. For between-group reconstruction, we built separate reconstruction models (F(*x_n_*) and F(*y_n_*)) from the dataset of each shape. Note that we implemented two basic experimental designs for drawn shapes and written characters separately. In general, we constructed four experimental designs.

### 2.2. Data Pre-Processing

During the training phase, the coordinates were collected during each trial every 50 ms and converted to the differences between the present state and the previous one. Simultaneously, based on the adjacent windowing techniques [26,27], sEMG signals were segmented for feature extraction every 50 ms. Then, we applied Root Mean Square (RMS) transformation to sEMG data to extract features every 50 ms. For a detailed illustration of data pre-processing, see [24].

### 2.3. Reconstruction Algorithm

Based on our previous study [24], we proposed a novel hybrid algorithm (KF-GEP). It deeply merges the Kalman Filter with the GEP for the sEMG-based drawing and handwriting trace reconstruction. The KF-GEP algorithm has two subroutines: the KF-GEP Parameters Training routine (Algorithm 1) and the Continuous Coordinate Reconstruction routine (Algorithm 2).

**Algorithm 1** KF-GEP Parameters Training
**Input:**
Training set $RMS_{i=1}^{k}$, *x*-coordinate sequence $x_{i=1}^{k}$, *y*-coordinate sequence $y_{i=1}^{k}$

**Output:**
State-transition model *A*, Process noise covariance *Q*, Covariance of the observation noise *R*, Observation model of $\Delta \hat{x}$: GEP_*X*_ ( ), Observation model of $\Delta \hat{y}$: GEP_*Y*_ ( )

**Initialize:**
Initial *x*-coordinate $x_{0}\leftarrow 0$; Initial *y*-coordinate $y_{0}\leftarrow 0$;Parameters involved in the GEP algorithm as shown in Table 1
1: 
**for**
i←1 to k
**do**
2:  $\Delta x_{i}\leftarrow x_{i}-x_{i-1}$3:  $\Delta y_{i}\leftarrow y_{i}-y_{i-1}$4:  $\Delta x_{i}^{\prime }\leftarrow \Delta x_{i+1}^{T}$5:  $\Delta y_{i}^{\prime }\leftarrow \Delta y_{i+1}^{T}$6:  $\Delta xy_{i}\leftarrow \left[\Delta {x_{i}}^{T},\Delta {y_{i}}^{T}\right]$7: 
**end for**
8:  $\langle \beta_{x},u\rangle \leftarrow \mathrm{BiLR}\left(\Delta xy,\;\Delta x^{\prime }\right)$9:  $\langle \beta_{y},v\rangle \leftarrow \mathrm{BiLR}\left(\Delta xy,\;\Delta y^{\prime }\right)$10:  $\langle {\mathrm{GEP}}_{X}\left(\;\right),\;u^{\prime }\rangle \leftarrow \mathrm{runGEP}\left(RMS,\Delta x\right)$11:  $\langle {\mathrm{GEP}}_{Y}\left(\;\right),\;v^{\prime }\rangle \leftarrow \mathrm{runGEP}\left(RMS,\Delta y\right)$12:  $A\leftarrow {\left[\beta_{x}^{T},\;\beta_{y}^{T}\right]}^{T}$13:  Q←cov(u, v)14:  $R\leftarrow \mathrm{cov}\left(u^{\prime },\;v^{\prime }\right)$

The KF-GEP Parameters Training routine is called with the predicting step in Kalman Filter. Where *A* is the [2 × 2] state-transition model matrix, *Q* is the process noise covariance. Note that in the Algorithm 1, Line 8 & 9, the binary linear regression (BiLR) is used to calculate the coefficients and residuals:

(1) $$\Delta x_{i+1}=a_{1}\Delta x_{i}+a_{2}\Delta y_{i}+u_{i}$$

(2) $$\Delta y_{i+1}=b_{1}\Delta x_{i}+b_{2}\Delta y_{i}+v_{i}$$

where $a_{1}$, $a_{2}$, $b_{1}$ and $b_{2}$ represent the coefficients, $u_{i}$ and $v_{i}$ represent residuals. Further, $\beta_{x}=\left[\begin{array}{cc} a_{1} & a_{2} \end{array}\right]$, $\beta_{y}=\left[\begin{array}{cc} b_{1} & b_{2} \end{array}\right]$, $A=\left[\begin{array}{cc} a_{1} & a_{2}\\ b_{1} & b_{2} \end{array}\right]$ and Q=cov(u, v) can be deduced (Algorithm 1, Lines 12 & 13).

Note that in the KF-GEP algorithm, the GEP is used to construct two non-linear observation models as shown in Algorithm 1, Lines 10 & 11:

(3) $$\Delta x_{i}={\mathrm{GEP}}_{X}\left(RMS_{i}\right)+u_{i}^{\prime }$$

(4) $$\Delta y_{i}={\mathrm{GEP}}_{Y}\left(RMS_{i}\right)+v_{i}^{\prime }$$

where GEP_*X*_ (*RMS*) and GEP_*Y*_ (*RMS*) respectively map the sEMG signals space into the *X*-coordinates space and the *Y*-coordinates space, $u_{i}^{\prime }$ and $v_{i}^{\prime }$ represent residuals and the covariance of the observation noise $R=\mathrm{cov}\left(u^{\prime },v^{\prime }\right)$ can be deduced (Algorithm 1, Line 14).

**Algorithm 2** Continuous Coordinate Reconstruction
**Input:**
Testing set $RMS_{t=1}^{n}$, Observation models GEP_*X*_ ( ) and GEP_*Y*_ ( ), KF parameters *A*, *Q*, *R*

**Output:**
Updated *x*-coordinate sequence $\hat{x}$; Updated *y*-coordinate sequence $\hat{y}$

**Initialize:**
Initial *x*-coordinate ${\hat{x}}_{0}\leftarrow 0$; Initial *y*-coordinate ${\hat{y}}_{0}\leftarrow 0$; Initial starting state $S_{0}\leftarrow 0$;Covariance matrix with suitable variances $P_{0}\leftarrow I_{2}$
1: 
**for**
t←1 to n
**do**
2:  ${\hat{S}}_{t}\leftarrow A\;S_{t-1}$3:  ${\hat{P}}_{t}\leftarrow A\;P_{t-1}A^{T}+Q$4:  $K_{t}\leftarrow {\hat{P}}_{t}\;{\left({\hat{P}}_{t}+R\right)}^{-1}$5:  $P_{t}\leftarrow {\hat{P}}_{t}-K_{t}{\hat{P}}_{t}$6:  $\Delta {\hat{x}}_{t}\leftarrow {\mathrm{GEP}}_{X}\left(RMS_{t}\right)$7:  $\Delta {\hat{y}}_{t}\leftarrow {\mathrm{GEP}}_{Y}\left(RMS_{t}\right)$8:  $C_{t}\leftarrow \left[\Delta {{\hat{x}}_{t}}^{T},\Delta {{\hat{y}}_{t}}^{T}\right]$9:  $S_{t}\leftarrow {\hat{S}}_{t}+K_{t}\left(C_{t}-{\hat{S}}_{t}\right)$10:  ${\hat{x}}_{t}\leftarrow S_{t}\left(1\right)$ + ${\hat{x}}_{t-1}$11:  ${\hat{y}}_{t}$
$\leftarrow S_{t}\left(2\right)$ + ${\hat{y}}_{t-1}$12: 
**end for**


After KF-GEP Parameters Training terminates, a set of metrics/functions, *A*, *Q*, *R*, GEP_*X*_ ( ) and GEP_*Y*_ ( ), are available for Continuous Coordinate Reconstruction routine, which is called with the update step in KF. Note that in the Algorithm 2, the measurement transformation matrix *H* via multivariate linear regression equation [3,24] is fully replaced by the $C_{t}=\left[\begin{array}{c} {\mathrm{GEP}}_{X}\;\left(RMS_{t}\right)\\ {\mathrm{GEP}}_{y}\;\left(RMS_{t}\right) \end{array}\right]$, as shown in Algorithm 2, Line 8.

To verify the validity and robustness of the algorithm, its performance will be compared with the use of GEP and KF separately. Reconstruction accuracy will be measured by the squared correlation coefficient (*R*^2^). The more detailed calculation process of KF, GEP and *R*^2^ can be found in Reference [24]. Finally, the paired sample Wilcoxon signed-rank test will be used to assess whether the reconstruction performance (*R*^2^s) of the KF-GEP do significantly tends to be better than those of the KF and the GEP.

## 3. Results

Table 1 shows various parameters used in the GEP per run. The parameter selection was the same as our previous paper [24] in order to compare the reconstruction performance between the GEP and the KF-GEP fairly. GeneXproTools 5.0 software was employed to develop the GEP models. The computing programs of the KF-GEP were written by Matlab in Windows 7 and run on a computer with a 2.8 G Intel Core I5 and 8 G RAM. We trained and tested the reconstruction models on the dataset across participants. The dataset was randomly divided into two subsets after feature extraction. 70% of the data were selected as the training set and 30% as the test set.

### 3.1. Results of the KF-GEP

#### 3.1.1. Within-Group Reconstruction for Drawing

**Figure 5:**
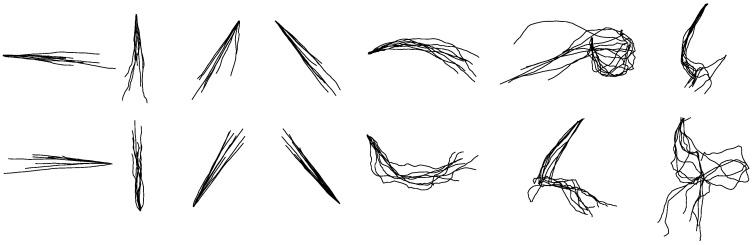
The randomly selected reconstructed drawing traces of with-group reconstruction by the KF-GEP. The shapes are, from left to right, in order, horizontal line, vertical line, forward slash, backslash, arch, circle, ellipse, reversed horizontal line, reversed vertical line, reversed forward slash, reversed backslash, reversed arch, square and triangle.

The reconstruction accuracies of each hand-drawn shape in terms of *R*^2^s are listed in Table 2. As shown in Table 2, the average accuracy reaches 0.68 ± 0.19 for reconstructed *X*-coordinates and 0.62 ± 0.12 for reconstructed *Y*-coordinates. Figure 5 shows the reconstruction results of each shape from one participant. The overlapping reconstructed traces shown in Figure 5 were randomly selected from test trials. Although there exist noise and inaccuracy, most of the reconstructed shapes are identifiable except for circle, ellipse, square and triangle.

#### 3.1.2. Within-Group Reconstruction for Drawing

**Figure 6:**
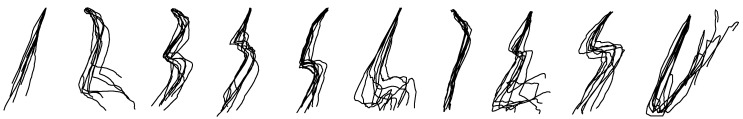
The randomly selected reconstructed numeric characters of with-group reconstruction by the KF-GEP.

The accuracies of each handwriting character are listed in Table 3. As shown in Table 3, the average reconstruction accuracy of *X*-coordinate is 0.40 ± 0.19 and that of *Y*-coordinate is 0.59 ± 0.06. Figure 6 shows the reconstruction results of each character from one participant. Most written numeric characters are identifiable despite the “4”, “6”, “8” and “9” are noisy and inaccurate.

#### 3.1.3. Between-Group Reconstruction for Drawing

**Figure 7:**
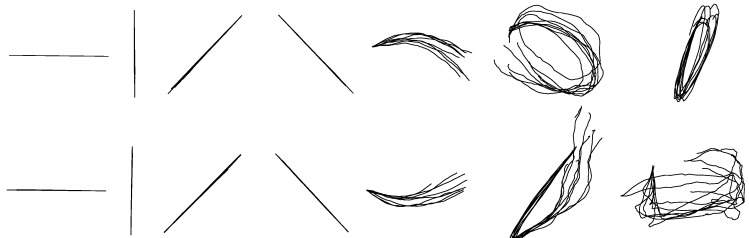
The randomly selected reconstructed drawing traces of between-group reconstruction by the KF-GEP.

The between-group reconstruction models of *X* and *Y* coordinates were built for each hand-drawn shape separately. Fourteen models of the *X*-coordinate and fourteen models of the *Y*-coordinate for each shape were built totally. Table 4 shows the average accuracies of each reconstructed shape based on separate KF-GEP models. As shown in Table 4, the average reconstruction accuracy of *X*-coordinate is 0.84 ± 0.13 and that of *Y*-coordinate is 0.77 ± 0.15, respectively higher than the results in within-group reconstruction. Figure 7 shows the reconstruction results of each shape from one participant. The overlapping reconstructed traces shown in Figure 7 were randomly selected from test trials. Compared with the unified models in within-group reconstruction, the separate models in between-group reconstruction for each hand-drawn shape perform better with a more accurate and specific prediction. This phenomenon is visually evident from the comparison of Figure 5 and Figure 7.

#### 3.1.4. Between-Group Reconstruction for Handwriting

**Figure 8:**
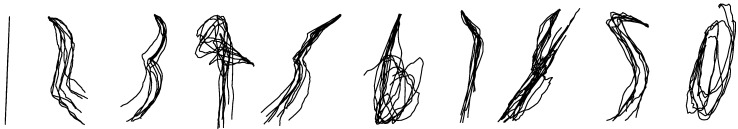
The randomly selected reconstructed numeric character of between-group reconstruction by the KF-GEP.

The between-group reconstruction models of *X* and *Y* coordinates were built for each numeric character separately. Ten models of the *X*-coordinate and Ten models of the *Y*-coordinate for each numeric character were built totally. Table 5 shows the average accuracies of each reconstructed character based on separate KF-GEP models. The average reconstruction accuracy of *X*-coordinate is 0.44 ± 0.18 and that of *Y*-coordinate is 0.78 ± 0.13, respectively higher than the results in within-group reconstruction. Figure 8 shows the reconstruction results of each character from one participant. The overlapping reconstructed traces shown in Figure 8 were randomly selected from test dataset. Compared with the unified models in within-group reconstruction, the separate models in between-group reconstruction for each numeric character perform better with a more accurate and specific prediction. This phenomenon is visually evident from the comparison of Figure 6 and Figure 8.

### 3.2. Results of the Kalman Filter

#### 3.2.1. Within-Group Reconstruction for Drawing

**Figure 9:**
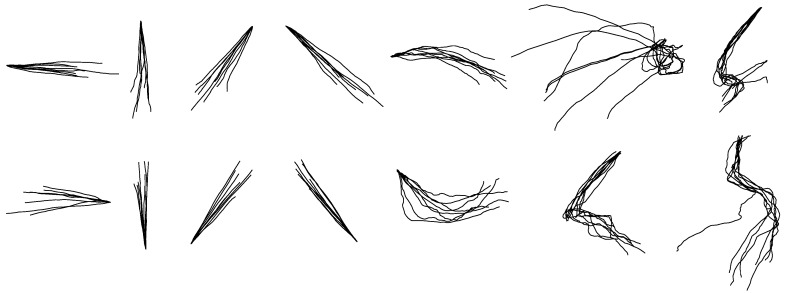
The randomly selected reconstructed drawing traces of within-group reconstruction by the KF.

The reconstruction accuracies of each hand-drawn shape attained by KF are listed in Table 6. The average accuracy is 0.65 ± 0.2 for reconstructed *X*-coordinates and 0.58 ± 0.1 for reconstructed *Y*-coordinates. Figure 9 shows the reconstruction results of each shape from one participant.

#### 3.2.2. Within-Group Reconstruction for Handwriting

**Figure 10:**
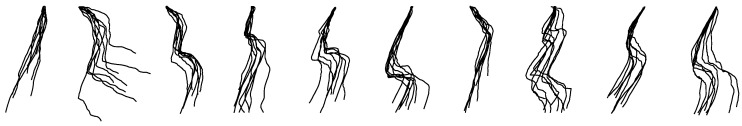
The randomly selected reconstructed numeric character of within-group reconstruction by the KF.

The accuracies of each handwriting character attained by the KF are listed in Table 7. The average reconstruction accuracy of *X*-coordinate is 0.26 ± 0.21 and that of *Y*-coordinate is 0.42 ± 0.03. Figure 10 shows the reconstruction results of each character from one participant. Only the “1”, “2”, “3” and “7” are identifiable.

#### 3.2.3. Between-Group Reconstruction for Drawing

**Figure 11:**
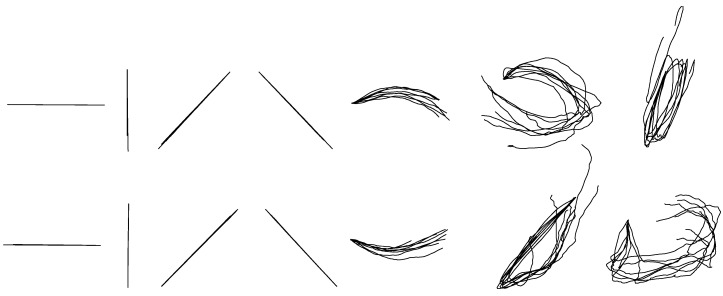
The randomly selected reconstructed drawing traces of between-group reconstruction by the KF.

Table 8 shows the average accuracies of each reconstructed shape based on separate KF models. The average reconstruction accuracy of *X*-coordinate is 0.79 ± 0.12 and that of *Y*-coordinate is 0.76 ± 0.15, respectively higher than the results in within-group reconstruction. Figure 11 shows the reconstruction results of each shape from one participant. Compared with the unified models in within-group reconstruction, the separate models in between-group reconstruction for each shape perform better.

#### 3.2.4. Between-Group Reconstruction for Drawing

**Figure 12:**
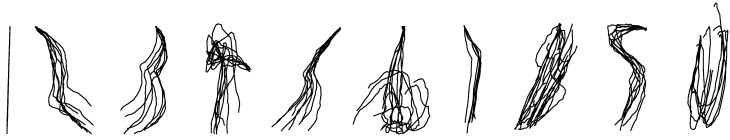
The randomly selected reconstructed numeric character of between-group reconstruction by the KF.

Table 9 shows the average accuracies of each reconstructed character based on separate KF models. The average reconstruction accuracy of *X*-coordinate is 0.30 ± 0.16 and that of *Y*-coordinate is 0.72 ± 0.13. Figure 12 shows the reconstruction results of each numeric character from one participant. Compared with the unified models in within-group reconstruction, the separate models in between-group reconstruction for each numeric character perform better but the “4”, “5”, “8” and “9” are still ambiguous.

### 3.3. Results of the GEP

#### 3.3.1. Within-Group Reconstruction for Drawing

**Figure 13:**
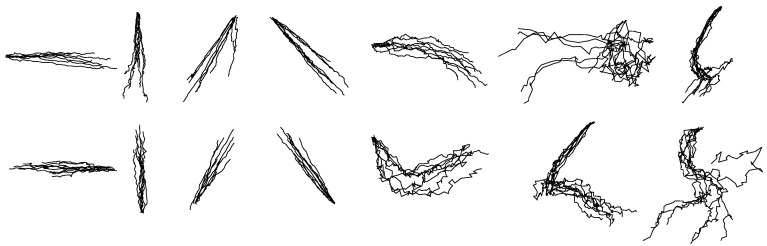
The randomly selected reconstructed drawing traces of within-group reconstruction by the GEP.

The reconstruction accuracies of each hand-drawn shape attained by the GEP are listed in Table 10. The average accuracy is 0.64 ± 0.2 for reconstructed *X*-coordinates and 0.61 ± 0.12 for reconstructed *Y*-coordinates. Figure 13 shows the reconstruction results of each shape from one participant. All the reconstructed shapes look serrated.

#### 3.3.2. Within-Group Reconstruction for Handwriting

**Figure 14:**
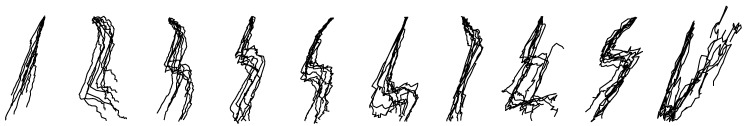
The randomly selected reconstructed numeric character of within-group reconstruction by the GEP.

The accuracies of each handwriting character attained by the GEP are listed in Table 11. The average reconstruction accuracy of *X*-coordinate is 0.37 ± 0.2 and that of *Y*-coordinate is 0.57 ± 0.08. Figure 14 shows the reconstruction results of each character from one participant. The “1”, “2”, “3”, “7” and “0” could be identified but the traces are not smooth.

#### 3.3.3. Between-Group Reconstruction for Drawing

**Figure 15:**
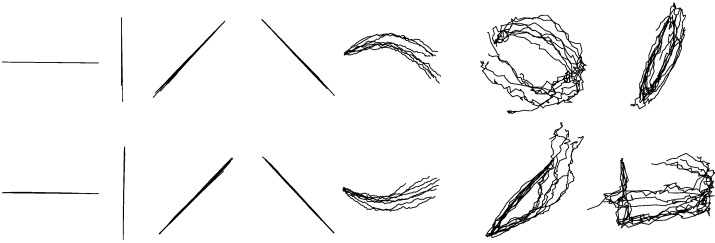
The randomly selected reconstructed drawing traces of between-group reconstruction by the GEP.

Table 12 shows the average accuracies of each reconstructed shape based on separate GEP models. The average reconstruction accuracy of *X*-coordinate is 0.82 ± 0.13 and that of *Y*-coordinate is 0.74 ± 0.17, respectively higher than the results in within-group reconstruction. Figure 15 shows the reconstruction results of each shape from one participant. Compared with the unified models in within-group reconstruction, the separate GEP models for each shape perform better.

#### 3.3.4. Between-Group Reconstruction for Handwriting

**Figure 16:**
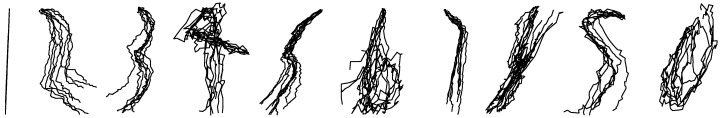
The randomly selected reconstructed numeric character of between-group reconstruction by the GEP.

Table 13 shows the average accuracies of each reconstructed character based on separate GEP models. The average reconstruction accuracy of *X*-coordinate is 0.39 ± 0.21 and that of *Y*-coordinate is 0.74 ± 0.13. Figure 16 shows the reconstruction results of each numeric character from one participant. Compared with the unified models in within-group reconstruction, the separate GEP models for each numeric character perform better but the “5”, “8” and “9” are still ambiguous and not smooth.

### 3.4. The Comparison of the Three Methods

**Figure 17:**
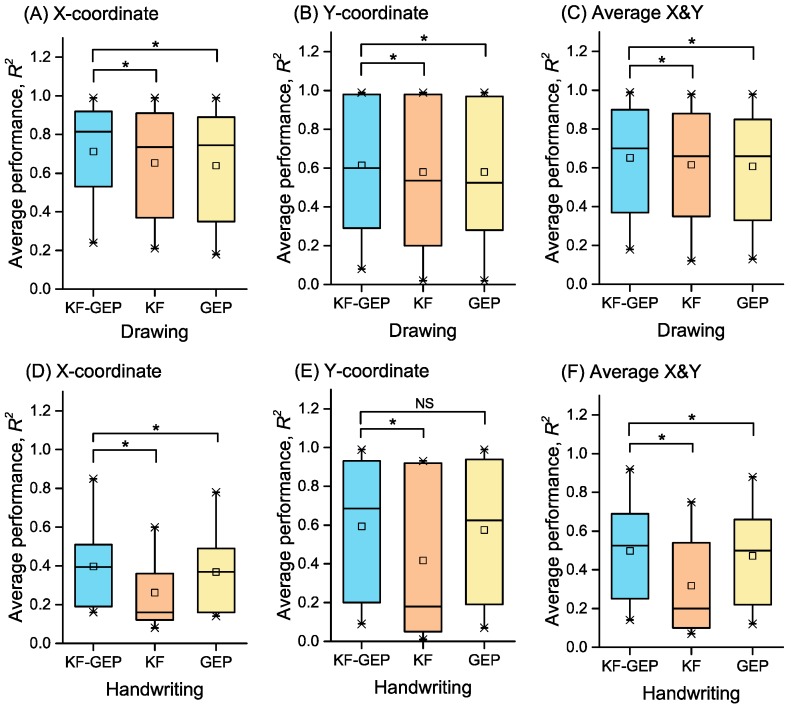
The averages of *R*^2^s in within-group reconstruction achieved by three methods. Statistical difference between the KF-GEP and KF and between the KF-GEP and GEP was measured by paired sample Wilcoxon signed-rank test: * the KF-GEP’s performance do significantly tends to be greater than the KF’s and GEP’s at the 0.05 level.

**Figure 18:**
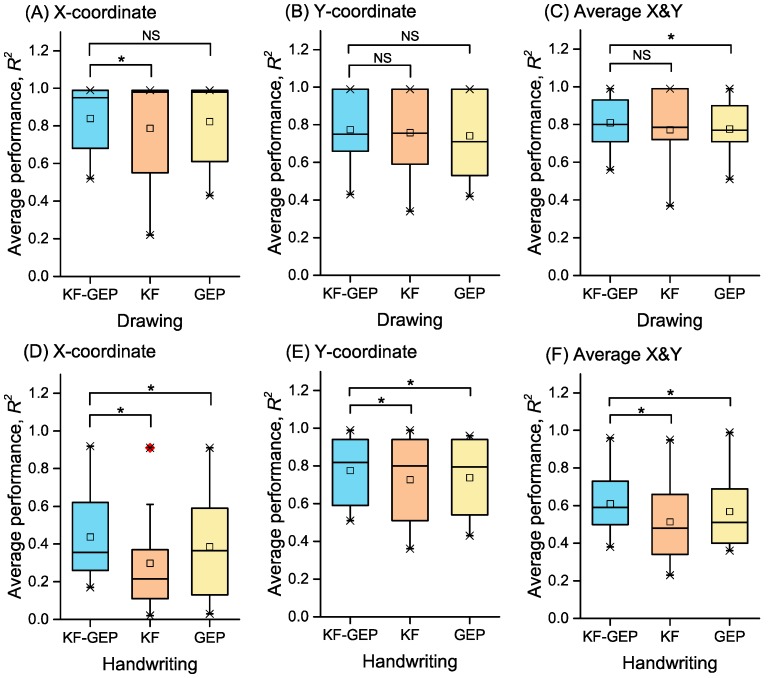
The averages of *R*^2^s in between-group reconstruction achieved by three methods. Statistical difference between the KF-GEP and KF and between the KF-GEP and GEP was measured by paired sample Wilcoxon signed-rank test: * the KF-GEP’s performance do significantly tends to be greater than the KF’s and GEP’s at the 0.05 level.

For each method, the performance of between-group reconstruction was better than that of within-group reconstruction. The result was similar to that of prior studies [3,24]. The averages of *R*^2^s of all hand-drawn shapes and hand-written characters across subjects achieved by the KF-GEP, the KF and the GEP in within-group reconstruction are respectively shown in Figure 17. Those in between-group reconstructions are respectively shown in Figure 18. The statistical difference between the average KF-GEP and the average KF, between the KF-GEP and the average GEP measured by the paired sample Wilcoxon signed-rank test can also be found in Figure 17 and Figure 18. The Figures directly show that compared to the KF and the GEP, the KF-GEP can always reach significantly higher accuracy at the 0.05 level for both within-group reconstruction and between-group reconstruction.

## 4. Discussion

In this paper, a novel hybrid algorithm which named KM-GEP fusing the GEP into the KF framework is applied to reconstructing hand-drawn and hand-written symbols based on dynamic sEMG activities. The study is the extension of our previous work which is the first to reconstruct *X* and *Y* coordinates from sEMG signals of arm muscles by the GEP [24]. The results of this study demonstrate that the proposed KF-GEP method performs very well.

In the performance comparison of three methods, the KF-GEP ranks first, GEP ranks second and the KF ranks worst. The measurement transformation matrix *H* of the KF models in this work were based on multivariate linear regression, mapping the sEMG features to the corresponding coordinates. Although the KF had the lowest prediction performance, it can smooth the noise so that obtain comprehensible and realistic results. The GEP algorithm has the advantage to develop simple explicit formulations with high accuracy [24,28,29]. Our GEP models generally outperformed the KF models because of the non-linear nature of the relation between the recorded sEMG features and the actuator traces. However, the symbols reconstructed by the GEP models looked rough and jagged because of the GEP algorithm without fusing the physical characteristics of drawing like the KF. The proposed KF-GEP models retained the predicting step and the updating step in the KF. The observation models GEP_*X*_ ( ) and GEP_*Y*_ ( ) were trained by the GEP algorithm and the role of the matrix *H* in the KF was substituted by them. In conclusion, the KF-GEP combines the advantages of the GEP and the KF, thus it can not only improve the reconstruction accuracy but also convert sEMG signals into smoother reconstructions of drawing traces.

Nevertheless, there are several limitations in this study:

(1) It can be found that the reconstruction accuracies of hand-written characters based on KF in this paper are worse than those in Reference [3]. The main reason may be that the experimental data used in our paper is different from that of [3]. We constructed the models across five subjects, however, Okorokova et al. (2015) constructed the models for 6 subjects separately. Thus, there always exists the inter-subject variety in our methods. The training and testing of models across the subjects are much more feasible, time-saving and natural for the on-line real-life scenario than that within each individual subject independently. In future work, we still need to explore a more advanced method suitable for different participants.
(2) The number of participants was relatively small and the dataset cannot represent the general population. However, the initial findings of this paper are worthy of further larger scale study. In future work, we will look at gender, health status, age and so on for the potential statistical variation.
(3) We pointed out that combining the KF with GEP can not only smooth the noise but also increase the reconstruction accuracy significantly. However, the average *R*^2^s attained from the KF-GEP are still below 0.85. In future work, we will try to use some other state-of-the-art deep-learning algorithms, such as Convolutional Neural Network, Recurrent Neural Network and so on, to improve the prediction performance.
(4) Compare to the results of our previous work [24], the average performance of all 14 shapes decreased. The main reason may be that the additional two shapes (triangle and rectangle) always have the lowest prediction performance. These two shapes are relatively complicated with turning during drawing. In addition, the reconstruction accuracies of the closed shapes (circle and ellipse) are still inferior. Thus, it can be concluded that this method can deduce reconstruction models for the basic one-stroke shapes which are not closed or complicated turnings but closed and complicated shapes can be formed with an arbitrary number of single strokes.

## 5. Conclusions

In this study, a hybrid algorithm KF-GEP was demonstrated to reconstruct the hand-drawn shapes and the hand-written numeric characters from the sEMG signal with encouraging performance results. The experimental results show that the KF-GEP models significantly outperform the separate KF models and GEP models. The proposed algorithm combines the advantages of KF and GEP, so it can not only smooth the noise but also develop non-linear models with high accuracy. In future work, the sample size for training should be enlarged and other state-of-the-art algorithms will be used to improve the reconstruction performance. The findings add to the literature on MuCI and can be introduced to many practical fields.

## Figures and Tables

**Table 1:** Parameters for the GEP algorithm in the training stage.

Parameter	Value
Number of chromosomes	200
Function set	F = {+, −, ×, /, *Sin*, *Cos*, *Sqrt*, *x*^2^, *Inv*, *Exp*}
Terminal	T = {*T*_1_, *T*_2_, *T*_3_, *T*_4_, *T*_5_, *T*_6_, *T*_7_}
Number of genes, head size, gene size	6, 15, 31
Linking function	Addition
Fitness function error type	RMSE
Mutation rate	0.00138
Inversion rate	0.00546
IS/RIS/gene transposition rate	0.00546
One-point/two-point recombination rate	0.00277
Gene recombination rate	0.00277

**Table 2:** Within-group reconstruction performance by the KF-GEP: the average accuracy of each shape across the five subjects.

Average Performance, *R*^2^			
Shape	*X*-Coordinate	*Y*-Coordinate	Average: (*X* + *Y*)/2
horizontal line	0.99 ± 0.00	0.71 ± 0.26	0.85 ± 0.13
vertical line	0.42 ± 0.27	0.95 ± 0.13	0.69 ± 0.15
forward slash	0.84 ± 0.25	0.99 ± 0.01	0.92 ± 0.10
backslash	0.99 ± 0.01	0.99 ± 0.01	0.99 ± 0.01
Arch	0.92 ± 0.08	0.49 ± 0.15	0.71 ± 0.11
Circle	0.24 ± 0.13	0.42 ± 0.28	0.33 ± 0.18
Ellipse	0.34 ± 0.20	0.09 ± 0.05	0.22 ± 0.15
Reversed horizontal line	0.88 ± 0.27	0.47 ± 0.32	0.68 ± 0.21
Reversed vertical line	0.53 ± 0.31	0.97 ± 0.06	0.75 ± 0.18
Reversed forward slash	0.81 ± 0.26	0.98 ± 0.01	0.90 ± 0.13
Reversed backslash	0.95 ± 0.16	0.99 ± 0.01	0.97 ± 0.08
Reversed arch	0.82 ± 0.25	0.29 ± 0.21	0.56 ± 0.18
Square	0.55 ± 0.30	0.19 ± 0.12	0.37 ± 0.20
Triangle	0.28 ± 0.12	0.08 ± 0.05	0.18 ± 0.08
All	0.68 ± 0.19	0.62 ± 0.12	0.65 ± 0.14

**Table 3:** Within-group reconstruction performance by the KF-GEP: the average accuracy of each numeric character across the five subjects.

Average Performance, *R*^2^			
Character	*X*-Coordinate	*Y*-Coordinate	Average: (*X* + *Y*)/2
“0”	0.49 ± 0.27	0.45 ± 0.26	0.47 ± 0.27
“1”	0.85 ± 0.12	0.99 ± 0.01	0.92 ± 0.07
“2”	0.44 ± 0.28	0.93 ± 0.02	0.69 ± 0.15
“3”	0.21 ± 0.21	0.95 ± 0.01	0.58 ± 0.11
“4”	0.19 ± 0.18	0.09 ± 0.02	0.14 ± 0.09
“5”	0.35 ± 0.22	0.93 ± 0.02	0.64 ± 0.13
“6”	0.16 ± 0.11	0.15 ± 0.10	0.16 ± 0.09
“7”	0.51 ± 0.17	0.92 ± 0.04	0.72 ± 0.10
“8”	0.18 ± 0.18	0.32 ± 0.19	0.25 ± 0.18
“9”	0.59 ± 0.23	0.20 ± 0.16	0.40 ± 0.19
All	0.40 ± 0.19	0.59 ± 0.06	0.50 ± 0.13

**Table 4:** Between-group reconstruction performance by the KF-GEP: the average accuracy of each shape across the five subjects.

Average Performance, *R*^2^			
Shape	*X*-Coordinate	*Y*-Coordinate	Average: (*X* + *Y*)/2
horizontal line	0.99 ± 0.002	0.78 ± 0.26	0.89 ± 0.15
vertical line	0.52 ± 0.26	0.99 ± 0.01	0.76 ± 0.12
forward slash	0.92 ± 0.27	0.93 ± 0.2	0.93 ± 0.25
backslash	0.99 ± 0.01	0.99 ± 0.004	0.99 ± 0.01
Arch	0.98 ± 0.01	0.68 ± 0.24	0.83 ± 0.12
Circle	0.79 ± 0.26	0.72 ± 0.28	0.76 ± 0.26
Ellipse	0.68 ± 0.28	0.69 ± 0.22	0.69 ± 0.25
Reversed horizontal line	0.99 ± 0.01	0.54 ± 0.34	0.77 ± 0.16
Reversed vertical line	0.71 ± 0.29	0.99 ± 0.01	0.85 ± 0.18
Reversed forward slash	0.99 ± 0.01	0.99 ± 0.004	0.99 ± 0.01
Reversed backslash	0.99 ± 0.004	0.99 ± 0.01	0.99 ± 0.004
Reversed arch	0.98 ± 0.01	0.43 ± 0.26	0.71 ± 0.02
Square	0.66 ± 0.32	0.46 ± 0.20	0.56 ± 0.28
Triangle	0.56 ± 0.22	0.66 ± 0.12	0.61 ± 0.16
All	0.84 ± 0.13	0.77 ± 0.15	0.81 ± 0.13

**Table 5:** Between-group reconstruction performance by the KF-GEP: the average accuracy of each numeric character across the five subjects.

Average Performance, *R*^2^			
Character	*X*-Coordinate	*Y*-Coordinate	Average: (*X* + *Y*)/2
“0”	0.73 ± 0.24	0.72 ± 0.27	0.73 ± 0.21
“1”	0.92 ± 0.06	0.99 ± 0.003	0.96 ± 0.03
“2”	0.28 ± 0.18	0.93 ± 0.01	0.61 ± 0.09
“3”	0.26 ± 0.06	0.97 ± 0.01	0.62 ± 0.04
“4”	0.32 ± 0.26	0.54 ± 0.26	0.43 ± 0.21
“5”	0.19 ± 0.06	0.94 ± 0.04	0.57 ± 0.03
“6”	0.39 ± 0.27	0.64 ± 0.29	0.52 ± 0.24
“7”	0.62 ± 0.25	0.92 ± 0.04	0.77 ± 0.14
“8”	0.17 ± 0.12	0.59 ± 0.25	0.38 ± 0.16
“9”	0.49 ± 0.29	0.51 ± 0.14	0.50 ± 0.15
All	0.44 ± 0.18	0.78 ± 0.13	0.60 ± 0.12

**Table 6:** Within-group reconstruction performance by the KF: the average accuracy of each shape across the five subjects.

Average Performance, *R*^2^			
Shape	*X*-Coordinate	*Y*-Coordinate	Average: (*X* + *Y*)/2
horizontal line	0.99 ± 0.07	0.64 ± 0.33	0.81 ± 0.17
vertical line	0.37 ± 0.29	0.98 ± 0.03	0.67 ± 0.14
forward slash	0.95 ± 0.08	0.98 ± 0.02	0.97 ± 0.04
backslash	0.99 ± 0.01	0.98 ± 0.05	0.98 ± 0.02
Arch	0.91 ± 0.07	0.38 ± 0.15	0.65 ± 0.09
Circle	0.21 ± 0.22	0.35 ± 0.15	0.28 ± 0.12
Ellipse	0.36 ± 0.16	0.03 ± 0.05	0.19 ± 0.08
Reversed horizontal line	0.84 ± 0.27	0.43 ± 0.31	0.63 ± 0.22
Reversed vertical line	0.48 ± 0.34	0.98 ± 0.03	0.73 ± 0.17
Reversed forward slash	0.77 ± 0.27	0.98 ± 0.01	0.88 ± 0.14
Reversed backslash	0.82 ± 0.28	0.99 ± 0.01	0.91 ± 0.14
Reversed arch	0.70 ± 0.3	0.20 ± 0.16	0.45 ± 0.18
Square	0.53 ± 0.35	0.17 ± 0.07	0.35 ± 0.18
Triangle	0.21 ± 0.15	0.02 ± 0.03	0.12 ± 0.08
All	0.65 ± 0.2	0.58 ± 0.10	0.62 ± 0.13

**Table 7:** Within-group reconstruction performance by the KF: the average accuracy of each numeric character across the five subjects.

Average Performance, *R*^2^			
Character	*X*-Coordinate	*Y*-Coordinate	Average: (*X* + *Y*)/2
“0”	0.36 ± 0.28	0.01 ± 0.01	0.19 ± 0.14
“1”	0.60 ± 0.35	0.01 ± 0.01	0.19 ± 0.14
“2”	0.15 ± 0.23	0.92 ± 0.03	0.54 ± 0.12
“3”	0.08 ± 0.17	0.93 ± 0.02	0.50 ± 0.08
“4”	0.16 ± 0.15	0.08 ± 0.04	0.12 ± 0.09
“5”	0.30 ± 0.24	0.93 ± 0.02	0.61 ± 0.08
“6”	0.12 ± 0.14	0.07 ± 0.05	0.09 ± 0.08
“7”	0.60 ± 0.17	0.89 ± 0.03	0.75 ± 0.10
“8”	0.09 ± 0.18	0.05 ± 0.05	0.07 ± 0.09
“9”	0.16 ± 0.17	0.28 ± 0.07	0.21 ± 0.12
All	0.26 ± 0.21	0.42 ± 0.03	0.28 ± 0.10

**Table 8:** Between-group reconstruction performance by the KF: the average accuracy of each shape across the five subjects.

Average Performance, *R*^2^			
Shape	*X*-Coordinate	*Y*-Coordinate	Average: (*X* + *Y*)/2
horizontal line	0.99 ± 0.002	0.77 ± 0.25	0.88 ± 0.12
vertical line	0.45 ± 0.25	0.99 ± 0.004	0.72 ± 0.12
forward slash	0.99 ± 0.007	0.99 ± 0.006	0.99 ± 0.006
backslash	0.99 ± 0.004	0.99 ± 0.004	0.99 ± 0.004
Arch	0.98 ± 0.01	0.65 ± 0.30	0.81 ± 0.15
Circle	0.76 ± 0.26	0.74 ± 0.26	0.75 ± 0.17
Ellipse	0.55 ± 0.25	0.59 ± 0.23	0.57 ± 0.14
Reversed horizontal line	0.99 ± 0.007	0.47 ± 0.33	0.73 ± 0.16
Reversed vertical line	0.59 ± 0.33	0.99 ± 0.007	0.79 ± 0.16
Reversed forward slash	0.99 ± 0.003	0.99 ± 0.004	0.99 ± 0.003
Reversed backslash	0.99 ± 0.003	0.99 ± 0.003	0.99 ± 0.003
Reversed arch	0.98 ± 0.01	0.59 ± 0.31	0.78 ± 0.16
Square	0.55 ± 0.36	0.34 ± 0.28	0.45 ± 0.22
Triangle	0.22 ± 0.21	0.52 ± 0.17	0.37 ± 0.10
All	0.79 ± 0.12	0.76 ± 0.15	0.77 ± 0.11

**Table 9:** Between-group reconstruction performance by the KF: the average accuracy of each numeric character across the five subjects.

Average Performance, *R*^2^			
Character	*X*-Coordinate	*Y*-Coordinate	Average: (*X* + *Y*)/2
“0”	0.61 ± 0.29	0.71 ± 0.29	0.66 ± 0.19
“1”	0.91 ± 0.06	0.99 ± 0.002	0.95 ± 0.03
“2”	0.02 ± 0.03	0.92 ± 0.01	0.47 ± 0.02
“3”	0.35 ± 0.24	0.96 ± 0.01	0.66 ± 0.12
“4”	0.21 ± 0.20	0.47 ± 0.24	0.34 ± 0.18
“5”	0.03 ± 0.02	0.94 ± 0.01	0.49 ± 0.01
“6”	0.15 ± 0.18	0.51 ± 0.30	0.33 ± 0.16
“7”	0.37 ± 0.32	0.89 ± 0.03	0.63 ± 0.16
“8”	0.11 ± 0.10	0.36 ± 0.25	0.23 ± 0.13
“9”	0.22 ± 0.23	0.52 ± 0.16	0.37 ± 0.14
All	0.30 ± 0.16	0.72 ± 0.13	0.51 ± 0.11

**Table 10:** Within-group reconstruction performance by the GEP: the average accuracy of each shape across the five subjects.

Average Performance, *R*^2^			
Shape	*X*-Coordinate	*Y*-Coordinate	Average: (*X* + *Y*)/2
horizontal line	0.99 ± 0.01	0.59 ± 0.32	0.79 ± 0.16
vertical line	0.35 ± 0.29	0.94 ± 0.13	0.65 ± 0.15
forward slash	0.76 ± 0.29	0.97 ± 0.06	0.88 ± 0.14
backslash	0.97 ± 0.03	0.99 ± 0.01	0.98 ± 0.02
Arch	0.91 ± 0.14	0.43 ± 0.17	0.67 ± 0.11
Circle	0.18 ± 0.13	0.34 ± 0.27	0.26 ± 0.16
Ellipse	0.23 ± 0.18	0.04 ± 0.06	0.13 ± 0.09
Reversed horizontal line	0.89 ± 0.30	0.46 ± 0.28	0.63 ± 0.23
Reversed vertical line	0.45 ± 0.32	0.97 ± 0.04	0.71 ± 0.17
Reversed forward slash	0.73 ± 0.28	0.98 ± 0.03	0.85 ± 0.14
Reversed backslash	0.89 ± 0.14	0.99 ± 0.01	0.94 ± 0.07
Reversed arch	0.81 ± 0.24	0.28 ± 0.22	0.55 ± 0.17
Square	0.55 ± 0.33	0.12 ± 0.10	0.33 ± 0.18
Triangle	0.23 ± 0.16	0.02 ± 0.03	0.13 ± 0.08
All	0.64 ± 0.20	0.61 ± 0.12	0.58 ± 0.14

**Table 11:** Within-group reconstruction performance by the GEP: the average accuracy of each numeric character across the five subjects.

Average Performance, *R*^2^			
Character	*X*-Coordinate	*Y*-Coordinate	Average: (*X* + *Y*)/2
“0”	0.49 ± 0.28	0.38 ± 0.24	0.44 ± 0.19
“1”	0.78 ± 0.19	0.99 ± 0.01	0.88 ± 0.09
“2”	0.42 ± 0.29	0.94 ± 0.02	0.68 ± 0.15
“3”	0.17 ± 0.26	0.95 ± 0.02	0.56 ± 0.13
“4”	0.16 ± 0.15	0.07 ± 0.04	0.12 ± 0.08
“5”	0.32 ± 0.22	0.93 ± 0.02	0.63 ± 0.12
“6”	0.14 ± 0.12	0.13 ± 0.08	0.13 ± 0.07
“7”	0.46 ± 0.08	0.87 ± 0.09	0.66 ± 0.04
“8”	0.16 ± 0.16	0.29 ± 0.17	0.22 ± 0.12
“9”	0.59 ± 0.21	0.19 ± 0.17	0.40 ± 0.16
All	0.37 ± 0.20	0.57 ± 0.08	0.47 ± 0.12

**Table 12:** Between-group reconstruction performance by the GEP: the average accuracy of each shape across the five subjects.

Average Performance, *R*^2^			
Shape	*X*-Coordinate	*Y*-Coordinate	Average: (*X* + *Y*)/2
horizontal line	0.99 ± 0.003	0.74 ± 0.27	0.87 ± 0.14
vertical line	0.43 ± 0.26	0.99 ± 0.004	0.71 ± 0.13
forward slash	0.99 ± 0.01	0.93 ± 0.22	0.96 ± 0.10
backslash	0.99 ± 0.004	0.99 ± 0.004	0.99 ± 0.004
Arch	0.98 ± 0.01	0.68 ± 0.28	0.83 ± 0.14
Circle	0.78 ± 0.24	0.65 ± 0.31	0.71 ± 0.18
Ellipse	0.61 ± 0.24	0.46 ± 0.22	0.54 ± 0.12
Reversed horizontal line	0.99 ± 0.01	0.47 ± 0.33	0.73 ± 0.17
Reversed vertical line	0.59 ± 0.32	0.99 ± 0.01	0.79 ± 0.16
Reversed forward slash	0.99 ± 0.003	0.99 ± 0.003	0.9 ± 0.003
Reversed backslash	0.99 ± 0.003	0.99 ± 0.003	0.99 ± 0.003
Reversed arch	0.98 ± 0.01	0.53 ± 0.32	0.75 ± 0.16
Square	0.59 ± 0.34	0.42 ± 0.26	0.51 ± 0.23
Triangle	0.61 ± 0.19	0.55 ± 0.15	0.58 ± 0.11
All	0.82 ± 0.13	0.74 ± 0.17	0.78 ± 0.13

**Table 13:** Between-group reconstruction performance by the GEP: the average accuracy of each numeric character across the five subjects.

Average Performance, *R*^2^			
Character	*X*-Coordinate	*Y*-Coordinate	Average: (*X* + *Y*)/2
“0”	0.67 ± 0.27	0.67 ± 0.29	0.67 ± 0.19
“1”	0.91 ± 0.06	0.95 ± 0.03	0.99 ± 0.002
“2”	0.11 ± 0.16	0.94 ± 0.01	0.53 ± 0.08
“3”	0.42 ± 0.22	0.96 ± 0.01	0.69 ± 0.10
“4”	0.35 ± 0.29	0.43 ± 0.25	0.39 ± 0.17
“5”	0.03 ± 0.06	0.94 ± 0.01	0.49 ± 0.03
“6”	0.26 ± 0.32	0.54 ± 0.29	0.40 ± 0.21
“7”	0.59 ± 0.26	0.92 ± 0.03	0.75 ± 0.13
“8”	0.13 ± 0.12	0.59 ± 0.27	0.36 ± 0.15
“9”	0.38 ± 0.30	0.43 ± 0.14	0.41 ± 0.13
All	0.39 ± 0.21	0.74 ± 0.13	0.56 ± 0.12
